# Basophil sensitivity through CD63 or CD203c is a functional measure for specific immunotherapy

**DOI:** 10.1186/1476-7961-8-2

**Published:** 2010-02-16

**Authors:** Susan Mikkelsen, Bo Martin Bibby, Mette Konow Bøgebjerg Dolberg, Ronald Dahl, Hans Jürgen Hoffmann

**Affiliations:** 1 Department of Respiratory Diseases, Aarhus University Hospital, Aarhus, Denmark; 2 Department of Biostatistics, Faculty of Medicine, University of Aarhus, Aarhus, Denmark; 3 Clinical Institute, Faculty of Medicine, University of Aarhus, Aarhus, Denmark

## Abstract

**Background:**

Subcutaneous Immunotherapy (SCIT) modifies the allergic response and relieves allergic symptoms. SCIT is the only and a very effective treatment for insect venom allergy. We hypothesized that basophil sensitivity, measured through the basophil activation test, would decrease during SCIT up dosing. Expression of CD203c was compared to CD63 as marker for basophil activation, using a Bland Altman plot and ROC curves.

**Methods:**

Patients (n = 18) starting subcutaneous SCIT for wasp allergy with an up dosing scheme of 7 to 11 weeks were enrolled. Heparinised blood samples were drawn at weeks 1-4, 7 and at the first maintenance visit. Basophils were stimulated at 7 log dilutions of *V. vespula* allergen for 15 min, and were stained with CD203c and CD63. Basophils were identified as CD203c^+ ^leukocytes, and the proportion of CD63^+ ^and CD203c^+ ^cells were plotted against allergen concentration. A sigmoid curve was fitted to the points, and the allergen concentration at which half of the maximal activation was achieved, LC50, was calculated. In another series of experiments, LC50 calculated in whole blood (AP) was subtracted from LC50 calculated with basophils suspended in plasma from a nonatopic donor (HS) to determine the protective effect of soluble factors in blood of patients treated with SCIT.

**Results:**

Heparin blood basophil activation was similar through CD63 and CD203c. Basophils were significantly more sensitized three weeks after initiation of SCIT compared to baseline (p < 0,01). The difference in LC50 increased by 1,04 LC50 units (p = 0,04) in patients that had just achieved maintenance dose compared with patients before initiating SCIT. When maintenance allergen concentrations had been reached, an increase in the protective plasma component was documented. Blood basophil concentration was marginally reduced by SCIT.

**Conclusion:**

Basophil activation is a versatile and sensitive tool that measures changes in the humoral immune response to allergen during SCIT.

## Introduction

Allergy to insect venom can be life threatening, and leaves patients in a continuous state of anxiety [1]. Subcutaneous immunotherapy (SCIT) is able to modify the course of allergic disease, and is the only form of protection available against this allergy [2]. The efficacy of SCIT for venom allergy has been documented in a meta analysis [3]. SCIT induces cellular increase in IL-10 [4] and changes in the humeral immune system; a transient increase in free allergen specific immunoglobulin IgX (mainly, but not exclusively IgG4 and IgA) and a decrease in the ratio of free allergen specific immunoglobulin to allergen specific IgE bound to effector cells (mast cells and basophil granulocytes) [4,5].

The basophil activation test (BAT) is an increasingly attractive method of assessing a type I allergic response [6]. BAT has been used to measure effects of SCIT [7-9]. Although it may also have diagnostic potential, its real power emerges when it is used to determine basophil sensitivity with serial dilutions of allergen, where it correlates well with clinical symptom strength [5,10]. The optimal choice of activation marker has been a recurrent theme; CD63 has historical value [11], and CD203c has potential because it could be used for identification and as activation marker at the same time [12]. In addition to the blood basophils' attractiveness as surrogate marker for mast cell responses, it is a leukocyte that is recruited from blood to inflamed tissue in a type I allergic response [13,14]. Changes in blood basophil concentration during allergen provocation may be a useful objective marker for a type I allergic response [14].

We devised an objective method to measure basophil sensitivity, and hypothesised that we could use it to measure changes in basophil sensitivity and soluble Immunoglobulin induced in plasma by SCIT during the up dosing phase of SCIT for wasp venom.

## Methods

### Patients

The ethics committee of Aarhus County approved a project, in which patients aged 18 or older, registered to begin subcutaneous immunotherapy with Vespula Alutard (ALK-Abelló, Hørsholm, Denmark) were recruited. From January to April 2007, 20 patients were recruited and 18 patients completed the project (median age 54 years, interquartile range (IQR) 44-61 years, 8 women, 10 men). The clinical history of wasp allergy was confirmed by skin prick test (SPT) (ALK Abelló, Hørsholm, Denmark) and by measuring specific IgE with the CAP system (Phadia, Copenhagen, Denmark), with a threshold of 0,35 kU/L. A SPT of >3 mm was considered positive. The median concentration of specific IgE to vespula venom was 3,01 kU/L, with an IQR of 1,53 - 7 kU/L. Some of the patients (n = 5) were also sensitized to bee venom with a median and range of 0,95 kU/L, ranging from 0,88 - 1,23 kU/L. One patient was negative for both wasp and bee venom in the CAP test, but had a positive prick test for wasp. In SCIT increasing doses of an allergen, the patient is sensitized to, is injected subcutaneously once a week until a maintenance dose (prescheduled 100.000 SQU) is reached. Treatment continues for 3-5 years with injections every 6^th ^-8^th ^weeks. Allergen injections, in the up dosing phase, can be administered as clusters (11 injections at 7 visits) [15] or after the conventional regime with one injection at each visit (16 injections at 16 visits), if there is an increased risk of adverse effects to the treatment. It is possible to shift from the cluster up dosing regimen to the conventional. Six of the 16 patients that started on our cluster regimen completed it (Table 1). The remaining ten patients transferred to the conventional regimen, on which four patients had started. For generation of the ROC curve, additional patients (3 women, 3 men) with allergy to wasp venom (specific IgE > 0,35 kU/L), and healthy, stung controls (5 women, 4 men) that did not have specific IgE to wasp venom, were recruited. For determination of differences in LC50 (log10 of the allergen concentration resulting in 50% activation of basophils) as the protective component in patient plasma (see below) at maintenance dose, additional patients receiving maintenance dose of SCIT for wasp venom allergy (n = 3) or birch pollen allergy (n = 5) were recruited (3 women, 5 men).

**Table 1:** Selected salient clinical data for each patient

Pt nr	Treatment	Side effects	maintenance dose	Ratio CD63 activation at 0,1/1
1	Traditional	fatigue	40000	**1,07**
2	traditional	weals	100000	**1,05**
3	traditional	weals	100000	0,82
4	traditional	weals, fatigue	100000	0,87
5	traditional, discontinued	heartbeat, nervousness	(20)	0,78
6	cluster, discontinued	itchy tongue	(1000)	0,78
7	cluster	hyperventilation	100000	0,65
8	cluster	none	100000	**0,95**
9	cluster	none	100000	0,66
10	cluster	weals, itch, fatigue	100000	0,91
11	cluster	weals	100000	0,87
12	cluster	none	100000	0,71
13	cluster	weals	60000	0,51
14	cluster	weals	100000	**1,22**
15	cluster	weals, fatigue	100000	**1,00**
16	change	weals, oedema	100000	0,66
17	change	weals, fatigue	100000	0,59
18	change	weals	100000	**2,09**
19	change	weals	100000	**1,36**
20	change	weals	100000	**1,13**

Side effects, maintenance dose and CD63 activation at 0,1 ug/ml venom/1 ug/ml venom are listed against SCIT regimen (traditional 16 week up-dosing protocol/7 week cluster up dosing protocol/change from 7 week to 16 week protocol) applied. Where the ratio of activation exceeds 0,95 it has been shown to be predictive of side effects [7] and is printed bold. Side effects were weals around the site of injection

### Basophil activation

**Figure 1:**
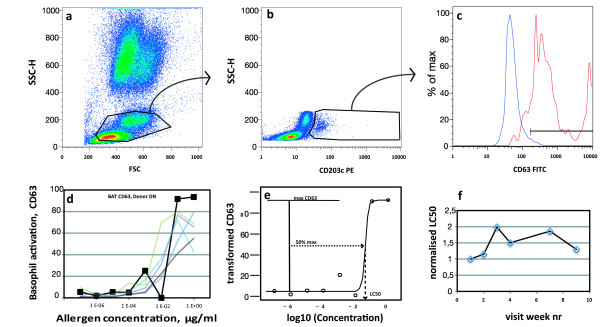
**Flow cytometric analysis of data of one representative patient**. a-c; gating of a representative experiment. Black regions and arrows indicate gating strategy. The PBS control is in blue, and the optimal allergen concentration (0,1 μg/ml) is in red. Histograms of expression of CD63 at visit 2 (d). Data smoothed by R for CD63 were used to determine LC50, the allergen concentration at which half-maximal activation (LC50) is achieved (e) LC50 was normalised to baseline and plotted against visit number.

For basophil activation, 4 ml venous blood was drawn into heparin tubes before allergen injection at visits 1, 2, 3, 4, 7 and at the first visit at which maintenance dose was injected. Basophil activation was done as previously described [6]; log dilutions of allergen (freeze-dried *V. vespula* allergen, a gift from ALK-Abello, Hørsholm, DK) ranging from 10 ug/ml final concentration to 10 pg/ml final concentration were compared with PBS only as control. Heparinised blood (100 ul) was added within 2 hours of venesection, and incubated with allergen at 37°C for 20 minutes. A dilution series of anti FceRI antibody CRA1 (Catalog Nr 334602, Biolegend, CA) was used as positive control (data not shown). Titrated amounts of CD63 FITC (5 ul, Invitrogen MHCD6301) and CD203c PE (5 ul, Beckmann-Coulter, IM3575) were added for the last 5 minutes. Activation was terminated by addition of 2 ml cold Saponin solution (0,15 mg/ml) in PBS for exactly 2 minutes, followed by 0,25 ml of 5% Methanol and 10% Paraformaledhyde in PBS and centrifugation at 400 g for 8 minutes at 4°C. Samples were washed once with PBS, suspended in PBS and 150 000 events were acquired on a FACS Calibur flow cytometer within 3 hours. On each day, compensation controls were acquired for CD63 FITC and CD203c PE with comp beads (Beckton-Dickinson, San Jose, CA). Data was compensated and in the analysis, the FS:SS region containing basophils was identified by back gating on CD203c^+ ^cells (figure 1). A threshold set at 2% positive cells in the PBS tube was used to determine percent activated cells in the tubes incubated with allergen. The fraction of positive cells is plotted against allergen concentration.

The protective effect of plasma on basophil activation was calculated as difference in LC50 in full blood (autologous plasma) and LC50 of basophils in washed blood. Washed blood was prepared by diluting 4 ml blood with 46 ml PBS, centrifuging and diluting again with PBS up to 50 ml. The remaining cells were suspended in heterologous plasma (1 ml) from a nonatopic donor for 15 min. Washed blood was used in a BAT test as described above. In a control experiment, the LC50 of washed blood was reconstituted with autologous plasma was similar to that of whole blood (n = 5).

LC50 was calculated in R [16] by fitting a sigmoid curve to data from each visit, and determining the amount of allergen, that results in 50% of maximal activation.

### Generation of ROC curve

The allergen concentration at which sensitivity and specificity were optimal for CD63 and for CD203c was determined with data from 24 clinically diagnosed wasp allergic patients and 9 stung controls with no specific IgE to vespula allergen, using plots of sensitivity and specificity [17], and confirmed with web based ROC software [18]. For SCIT patients, baseline visits were used for this analysis.

### Bland Altman Analysis

The difference in basophil activation as measured by up-regulation of CD63 and CD203c was investigated by a Bland Altman analysis [19].

### Blood basophil concentrations determined by constant volume acquisition (CVA)

Blood basophil concentration was determined by CVA [20]. Data is acquired for a constant, calibrated time, which appears to give a more precise measurement of volume than measurement with micro beads [20]. Briefly, 50 ul of blood was labelled with Lin1 FITC (BD Catalog Nr 340546), CD123 PE (BD Catalog Nr 340545), CD45 PE Cy7 (BD Catalog Nr 555484) and HLA DR APC (BD Catalog Nr 559866) for 15 min at room temp, and lysed with FACS Lyse (BD Catalog Nr 349202). Data was acquired for 4 minutes from all patients at all visits. Each day data was acquired, compensation controls were acquired, and data was compensated before analysis. Leukocytes were identified on a CD45 vs. SS plot. Basophils were identified as CD45^dim ^Lin1^neg ^CD123^bright ^HLA DR^neg ^cells, and the absolute concentration was calculated.

## Results

### Increase in basophil sensitivity during the up dosing phase of SCIT may be limited by factors in plasma

**Figure 2:**
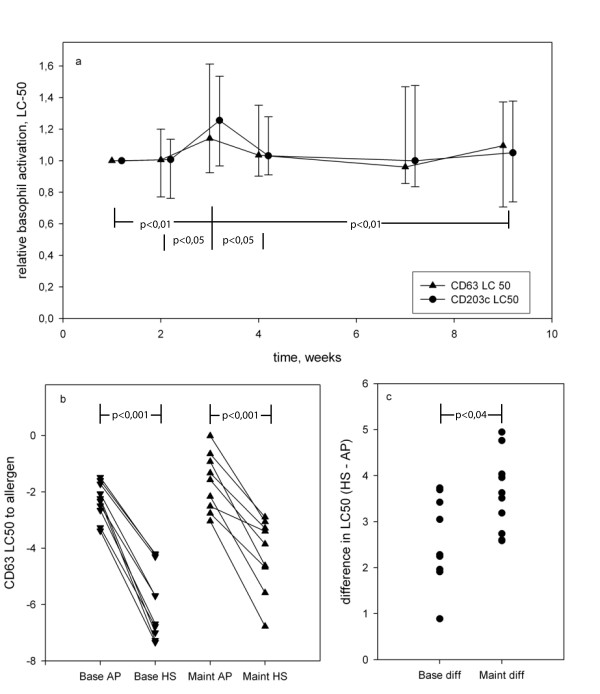
**The effect of SCIT on basophil activity**. (a) Median LC50 is plotted against visit for patients on SCIT (n = 18). At visit 3 basophils are significantly more sensitive than at other visits. (b) The protective effect of plasma is illustrated by replacing plasma with serum from a non-allergic donor. (c) The net protective effect measured as change in LC50 between basophils activated in autologous serum and in heterologous plasma is larger in patients on maintenance dose (n = 9) than in patients initiating SCIT (n = 10).

The effect of the up-dosing phase of subcutaneous immunotherapy on basophil up regulation of CD63 and CD203c (figure 1) was evaluated by fitting a sigmoid curve to data points obtained with a 7-step logarithmic dilution series of *V. vespula *allergen and blood of wasp allergic patients drawn at each of visits 1, 2, 3, 4, 7 and maintenance visit, if that did not coincide with visit 7. From this curve at each visit, the concentration of allergen resulting in half-maximal CD63 activation was determined (figure 1e). It is termed LC50. When normalised to the baseline visit, the LC50 was normally distributed. When plotted against visit, it increased significantly from the first to the third week of immunotherapy (visit 1 - 3; p = 0, 0098, visit 2 - 3 p = 0, 0259, visit 3 - 4 p = 0, 0274, visit 3-maint p = 0, 0095 for all in a mixed linear model, p = 0,029 for CD63, p = 0,015 for CD203c, n = 18), and tended toward baseline levels beyond that (figure 2a).

In a cross-sectional study to determine the contribution of humoral factors toward BAT performed in whole blood, blood from different patients initiating SCIT and patients on maintenance was washed with PBS and replaced by heterologous serum (termed HS in contrast to autologous plasma, AP) from a nonatopic donor as described in methods, and was used in a BAT. Replacing AP with HS dramatically increased basophil sensitivity. At baseline the sensitivity increased by 2,576 LC50 units (n = 8, p < 0,001), and at maintenance it increased by 3,595 LC50 units (n = 10, p < 0,001) (figure 2b). At the early stage of maintenance, the reduction in basophil sensitivity resulting from the autologous plasma had thus increased by 1,02 LC50 units (p = 0,04) (figure 2c).

### CD63 and CD203c are equivalent markers for basophil activation

**Figure 3:**
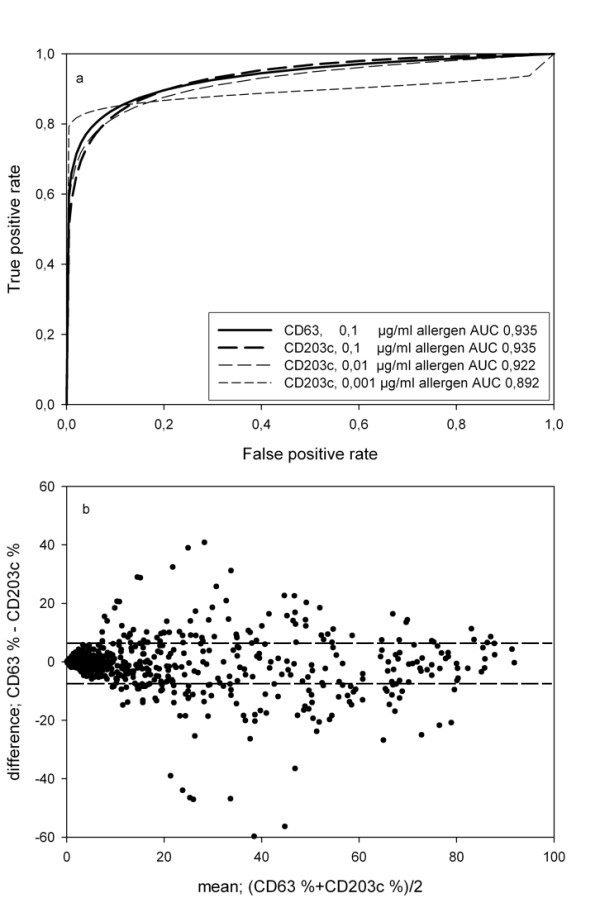
**Comparison of CD63 and CD203c as readout**. (a) the ROCs for CD63 and CD203c at the optimal concentration of allergen, 0,1 μg/ml, were indistinguishable. (b) Bland Altman plot of CD63-CD203c. Stippled lines indicate 25^th ^and 75^th ^quartiles.

The optimal allergen concentration used to detect allergy to *V. vespula *allergen was determined with sensitivity vs. specificity curves and is illustrated with a ROC curve (figure 3a) with 24 patients with documented allergy and 9 exposed controls. The optimal final *V. vespula* allergen concentration was 0,1 μg/ml. The sensitivity and specificity were 88% and 78% for CD63 at 3,28% basophil activation. For CD203c, the sensitivity and specificity were 88% and 89% at 3,85% basophil activation.

Agreement between activation measurements performed with CD63 as the predicate method and CD203c determined with a Bland Altman plot [19] (figure 3b). The median difference of CD63 - CD203c was -1,2%, IQR 23,5% to -28,6% (not normally distributed, n = 914).

Difference between CD63 and CD203c was determined with an approximate T test in a linear mixed model. For all concentrations there was no significant difference between CD63 and CD203c with all p-values greater than 0,11. When autologous plasma is replaced with heterologous serum, the value of CD63-CD203c increased from 4, 2% (IQR -20, 3 - 31, 7) to 41, 7% (IQR 8, 3 - 73, 5, n = 135. In the same linear mixed model that takes account for repeated measures the relative difference between activation through CD63 and CD203c was larger after plasma was replaced with serum (p = 0,0172). It increased significantly (n = 15, p < 0, 05) at four of the six highest allergen concentrations, suggesting that CD203c is up regulated less than CD63 when autologous plasma is replaced by heterologous serum. This was not due to an increase in baseline MFI for CD203c, as the baseline MFI for CD63 and CD203c increased 1,92 and 1,83 fold, respectively, after replacement of plasma with serum. This was not significant.

### The blood basophil concentration decreases marginally during SCIT

Blood basophil concentration measured using CVA was 37 (IQR 28 - 55, n = 18) cells/μl in allergic patients treated with SIT, and 35 (IQR 23 - 42, n = 6, p = 0, 06) cells/μl in controls. It increased by median 1 (IQR -2 - 6) cell/μl (5%) per 30 minutes for patients receiving allergen injections and by median 3 (IQR 2 - 5) cells/μl (12%) per 30 minutes in control persons after correction for time between sampling. The difference was significant in patients and controls when measured over 90 and 120 minutes (p < 0, 04), but not when measured over 30 minutes.

### Discordance between anamnesis, sIgE, prick test and BAT

On the scale proposed by Brown [21], 14 patients reported moderate symptoms and 4 patients reported severe symptoms when stung. Seven patients had other allergies (2 latex, 3 bee, 2 cat, 3 house dust mites, 1 perfume, 1 kiwi, 1 nuts, 1 apples) and other allergic disease (3 asthma, 3 hay fever, 3 Urticaria).

For two patients the activity of the basophils for both CD203c and CD63 was only just above the threshold values (i.e. positive) at some of the visits. These two patients had almost no symptoms/adverse effects to treatment with SIT, but they had moderate symptoms as response to wasp sting, specific IgE at 3,01 kU/L and 6,0 kU/L respectively and positive skin prick tests (SPT) before the beginning of SIT. One patient had moderate symptoms to wasp sting and positive skin prick test but negative specific IgE against wasp before the beginning of SIT. The BAT of this participant was positive at all visits, and the patient had local reactions to the treatment and a systemic reaction at visit 2.

## Discussion

We have monitored changes in basophil sensitivity as the logarithm of the concentration of allergen that elicits a half-maximal response, and blood basophil concentration, during the up dosing phase of SIT. We have found that there is a transient increase in basophil sensitivity after 3 weeks of SIT. This increase in sensitivity may reflect competing amounts of immunoglobulin on basophils (IgE) and free immunoglobulin in circulation (IgX, all classes of immunoglobulin with affinity for haptens on allergen). It coincides with the early decrease in basophil histamine release and increase in free IgG4 and IgA described recently [4].

During ultra rush procedures of SCIT with Japanese cedar [9] or birch or cat [8], maximal expression of CD203c as % activated cells on basophils [9] or MFI [8] after cross linking with allergen decreased with time. BAT could not predict results of a sting challenge at 0,1 or 1,0 ug venom [22]. In a 5-day rush up dosing regimen, BAT at suboptimal concentrations appeared elevated after 5 days corresponding to the findings after 3 weeks in the present 16-week regimen (the significance of this had not been tested). Basophil activation was significantly reduced after six months and after three years [23]. SCIT for birch or grass allergy significantly decreased CD-sens (analogous to LC50) tenfold [5], where the present results did not show a significant change. This may be because in the present experiment, BAT was done during the first 7 weeks assuming that all patients would complete up dosing by a semi rush protocol. Only 10 of 20 patients completed the semi-rush protocol. The protective component in plasma (that corresponds to Allergen Binding Activity (ABA)) increased by 1,04 LC50 units. This corresponds well to the tenfold change in ABA published recently [5].

Change in basophil activation measured as CD63 or CD203c activation at 0,1 ug/ml allergen did not vary significantly during the up dosing phase. A ratio of CD63 activation at 0,1 ug/ml/1 ug/ml > 92% has previously been shown to associate with severe side effects [7]. In this study, 8 patients had a ratio > 92%. This did not associate with side effects (Table 1). In a study of modified rush up dosing of insect venom, the ratio of basophil activation at 0,1 ug/ml venom/1 ug/ml venom above 92% at baseline predicted severe side effects during up dosing [7]. In the present study, 8 of 18 patients had a ratio of basophil activation predicting severe side effects. Neither others [24] nor we could confirm the usefulness of this ratio.

The up dosing scheme in the present study is much slower, and associated with very few side effects. This may explain that we do not find the decrease that was observed in the other studies, and why there were no side effects to associate with a high ratio of activation. The ratio of cell bound IgE to total free immunoglobulin may be the most significant effect parameter, which determines whether a basophil activation test becomes positive. The protective effect of SCIT in patient plasma (which we assume contains immunoglobulins competing for epitopes on allergen) had increased by >1 LC50 unit when comparing patients at baseline with patients just initiating maitenance therapy. Basophil activation has been shown to correlate well with clinical measures [10]. Our study did not extend long enough to demonstrate an effect of treatment on basophil activation by flow cytometry, which has been shown by others [5]. Inhibition of histamine release, the gold standard for basophil activity, was significantly different from baseline after 6 weeks of SCIT [4]. Whilst single concentration analyses of basophil activation may not be sufficient to monitor effects of SCIT [22], we confirm here that this can be done with logarithmic series of dilutions of allergen.

CD-sens is the fist algorithm developed to measure basophil sensitivity in a basophil activation test [25]. The allergen dose giving maximal activation is determined, and a line of regression is calculated through that point and the activation rates at the two preceding allergen concentrations. The half-maximal activation is determined from this interpolated line. CD-sens [25] was evaluated as a technique to calculate basophil sensitivity, but was not found to be useful in our hands because the allergen concentrations we used were too low to obtain a maximal activation as most of the curves we obtained were not bell shaped, and because our log dilutions were too widely spaced to obtain accurate CD-sens. LC50 considers more values than CD-sens, and may be less subjective. The values obtained with LC50 were larger than those obtained with CD-sens.

The present data gave us an opportunity to determine the optimal allergen concentration for diagnosing wasp allergy with a ROC curve to be 0,1 ug/ml final concentration in the reaction vessel with blood, which compares well with 0,5 ug/ml [22] and 0,5 - 0,05 ug/ml [23] final concentration of V vespula allergen in BAT published by others. The ROC curves for CD63 and CD203c and the threshold for positivity were almost identical for CD63 and CD203c, suggesting that the two markers measure similar aspects of basophil activation. Although Boumiza has disputed this [26], it has since been confirmed by a number of clinically oriented publications [24,27,28]. We have previously shown that choice of fluorochome and lysis procedure had favoured CD203c over CD63, illustrating the need to consider all aspects of an experiment [6]. We have done the first Bland Altman analysis of CD63 and CD203c to show that in full blood there is no difference between the markers, whereas there is significantly less up regulation of CD203c than of CD63 on washed basophils. The effect appears not to be due to increased baseline expression of CD203c, as this was similar for both markers. Washing away plasma immunoglobulins increases the sensitivity of the test significantly, but masks the clinical relevance as the total response is compounded from the free immunoglobulin absorption of allergen and the cross linking of cell bound IgE.

The basophil numbers we obtained using CVA were within a recently published reference interval [29]. Previously, we had shown that ingestion of allergen resulted in a 20% reduction of blood basophil concentration compared with placebo [14]. Allergen injection, as a negative control, resulted in a marginal reduction in blood basophil numbers compared with controls. Changes in blood basophil concentration may thus be a marker for type I allergic response. As the difference in blood concentration of basophils increased with time from 30 min to 120 min, the optimal time frame for measuring blood basophil concentration as marker for a type I allergic reaction remains to be determined.

Discordance between in vivo, ex vivo and in vitro tests is a recurrent problem in allergen diagnosis, and some have suggested that basophil activation should be the arbitrating test if anamnesis and specific IgE do not support the same conclusion [30]. We found a rate of discordance between patient history and in vitro diagnostic tests (3 patients of 18 with discordance between patient history and in vitro diagnostic tests) that was similar to others [23,30]. The fact that basophil activation in full blood correlates with clinical symptoms and does reflect the response by cell-bound IgE in the context of competing immunoglobulins argues that it may occupy a special niche amongst diagnostic tests for allergy.

## Conclusion

Basophil sensitivity through CD63 or Cd203c increases comparably during the first weeks of specific immunotherapy, but can measure a protective element already when maintenance dose is reached.

## Competing interests

The authors declare that they have no competing interests.

## Authors' contributions

SM did most experiments, analysed the data and wrote the first draft of the manuscript. MKBD did some experiments. BMB designed and performed statistical analyses. RD contributed to the study design and participated in analysis and writing. HJH conceived the study and wrote the manuscript. All authors have read and approved the final manuscript.
